# Attractive particle interaction forces and packing density of fine glass powders

**DOI:** 10.1038/srep06227

**Published:** 2014-09-02

**Authors:** Eric J. R. Parteli, Jochen Schmidt, Christina Blümel, Karl-Ernst Wirth, Wolfgang Peukert, Thorsten Pöschel

**Affiliations:** 1 Friedrich-Alexander-Universität Erlangen-Nürnberg, Germany; 2 Institute of Particle Technology (LFG), University of Erlangen-Nuremberg (FAU), Cauerstraße 4, D-91058 Erlangen, Germany

## Abstract

We study the packing of fine glass powders of mean particle diameter in the range (4–52) *μ*m both experimentally and by numerical DEM simulations. We obtain quantitative agreement between the experimental and numerical results, if both types of attractive forces of particle interaction, adhesion and non-bonded van der Waals forces are taken into account. Our results suggest that considering only viscoelastic and adhesive forces in DEM simulations may lead to incorrect numerical predictions of the behavior of fine powders. Based on the results from simulations and experiments, we propose a mathematical expression to estimate the packing fraction of fine polydisperse powders as a function of the average particle size.

The packing behavior of powders can be strongly influenced by inter-particle attractive forces of different types, such as adhesion and non-bonded van der Waals forces^1^,^2^,^3^,^4^,^5^,^6^. The relevance of the different types of attractive interactions for the packing density of powders of different materials and particle size distributions is, however, largely uncertain. That is, it is a challenging problem to *predict* the packing density of a certain granular system specified by the particle size distribution and the material properties of the particles^7^,^8^.

Numerical simulations by means of the Discrete Element Method (DEM)^9^,^10^ can offer a helpful tool in the investigation of the packing behavior of powders. In this type of numerical simulations, Newton's equation of motion is solved for all particles simultaneously by taking into account the forces and torques acting on each particle, both due to external fields and due to interactions with other particles in the system. However, in order to make reliable predictions of the behavior of the bulk from DEM simulations, an accurate physical modeling of the relevant forces governing the interactions between the particles is required.

Most previous studies of density of dry powder packings using DEM simulations were focused on monodisperse systems and included either van der Waals interactions^7^,^11^,^12^ or adhesive forces during contact^13^,^14^. Indeed, typical powders are poorly sorted and may contain a broad interval of particle sizes. For fine powders, the attractive interactions between particles of different sizes should have an important effect on the dynamics, since cohesive forces become increasingly relevant compared to gravitational forces as the particle size decreases^4^. Therefore, particle size distribution plays an essential role not only due to geometrical constraints^15^,^16^,^17^,^18^,^19^,^20^ but also because attractive forces have different influence for particles of different size. Consequently, the size distribution of particles and the adequate description of attractive forces acting on particles of different size should be taken into account in DEM simulations in order to yield a predictive description of fine powders.

The aim of our work is to provide numerical evidence that DEM simulations are able to describe the packing of fine powders correctly, that is in quantitative agreement with experiments, provided both the particle size distribution and the adequate model of attractive particle interaction are taken into account. We will show that this model should contain both relevant contributions due to adhesion (here modeled through JKR theory^21^) and van der Waals interactions. Neglecting any of these contributions leads for the case of fine powders to unacceptable deviations, that is the DEM method renders unreliable. We believe that this result, obtained for the packing of fine glass powders will be of relevance also for other systems, in particular, when the system contains a significant fraction of small particles or a wide distribution of particle sizes.

We characterize the particle size distribution by means of *q*_3_(*d*) ≡ d*Q*_3_(*d*)/d*d* where *Q*_3_(*d*) is the fraction of mass of all particles of diameter *d* or smaller, relating to the probability density of particle diameters, *p*(*d*), via 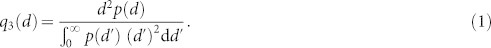
Figure 1 shows the size distribution, *q*_3_(*d*), of our experimental samples, each of them averaged over 5 independent measurements. For each sample, Tab. I provides the mean diameter, 
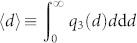
, and the obtained packing fraction, *φ*, again averaged over 5 independent measurements. Further, the corresponding 1%, 50%, and 99% quantiles, *d*_1,3_, *d*_50,3_, and *d*_99,3_, respectively, are given defined as 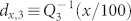, where 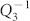 is the inverse of *Q*_3_(*d*). The values from Tab. I are used furtheron for reference in the subsequent figures.

We simulate the process using DEM, that is, simultaneously solving Newton's equations of translational and rotational motion for all particles. There is a variety of models to describe the contact forces in DEM simulation, suitable for different particle geometry and material behavior, for an overview see, e.g.^10^,^22^,^23^,^24^. In the present paper, we assume viscoelastic interaction in normal direction^25^ and apply a modified Cundall-Strack model^9^ for the tangential direction^26^. The corresponding forces read 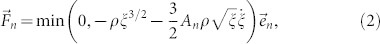
where 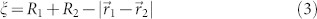
is the compression of particles of radii *R*_1_ and *R*_2_ at positions 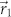 and 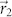, and 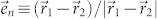 is the normal unit vector. The elastic parameter of Eq. (2), *ρ*, is a function of the Young's modulus, *Y*, the Poisson's ratio *ν*, and the effective radius *R*_eff_ ≡ *R*_1_*R*_2_/(*R*_1_ + *R*_2_), 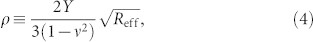
and the dissipative parameter, *A_n_*, depends, moreover, on the material viscosities. For details see^25^. While *ρ* can be computed directly from material characteristics which are easily available for a variety of materials, the viscosities needed for *A_n_* are not directly available. To determine *A_n_*, therefore, we use a relation between the coefficient of restitution, *ε*, for the collision of two isolated particles and *A_n_*^27^,^28^,^29^ where the Padé approximation from^30^ was employed.

The tangential force reads^26^

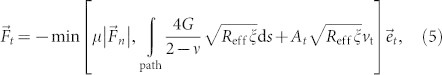
where *μ* is the Coulomb friction coefficient and *G* is the shear modulus, which is given by the equation, 2*G* = *Y*/(1 + *ν*). The integral in Eq. (5) is performed over the displacement of the particles at the point of contact for the duration of the contact^9^ and 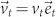 stands for the relative tangential velocity at the point of contact, where 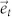 is the corresponding unit vector. The tangential dissipative parameter, *A_t_*, characterizes the surface roughness and is chosen such that the prefactors of the normal and tangential deformation rates (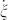 and *v*_t_) in Eqs. (2) and (5), respectively, are of the same order of magnitude^31^. Using this assumption, Ref. [32] found excellent agreement between simulation results and experimental values of particle velocity profiles in a gravity-driven shearing experiment. By comparing Eqs. (2) and (5), we obtain *A_t_* ≈ *A_n_Y*/(1 − *ν*^2^).

Adhesion is taken into account via the JKR model^14^,^21^,^33^,^34^

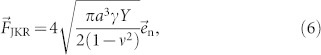
where *a* is the contact radius, related to the deformation *ξ* through 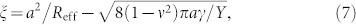
and *γ* is the surface energy density, which is a characteristic of the particle material^35^.

To compute the force, 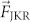, through Eq. (6), the contact radius, *a*(*ξ*) is determined from Eq. (7) as a function of the deformation *ξ*. Thus, Eq. (7) can be rewritten in the form^14^

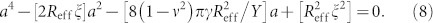
This equation can be solved analytically to obtain the contact radius, *a*, see Eq. (18).

For the case of fine powders, van der Waals force may have a non-negligible influence on the dynamics of the system. It is given by^36^,^37^

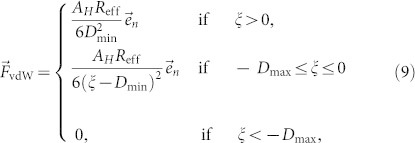
where *ξ* is given by Eq. (3). The Hamaker constant is related to the surface energy density via^2^

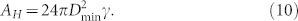
Furthermore, *D*_min_ is a parameter introduced to avoid the singularity of the Hamaker equation at *ξ* = 0. As a matter of fact, the surface of the particles is not smooth, such that there is always a minimal distance *D*_min_ between the particles at contact^38^,^39^. Here we take the value *D*_min_ = 1.65*Å*^2^,^39^. Finally, *D*_max_ is the maximal (cutoff) distance of the van der Waals interaction, which is set as 1 *μ*m.

## Results

**Figure 1:**
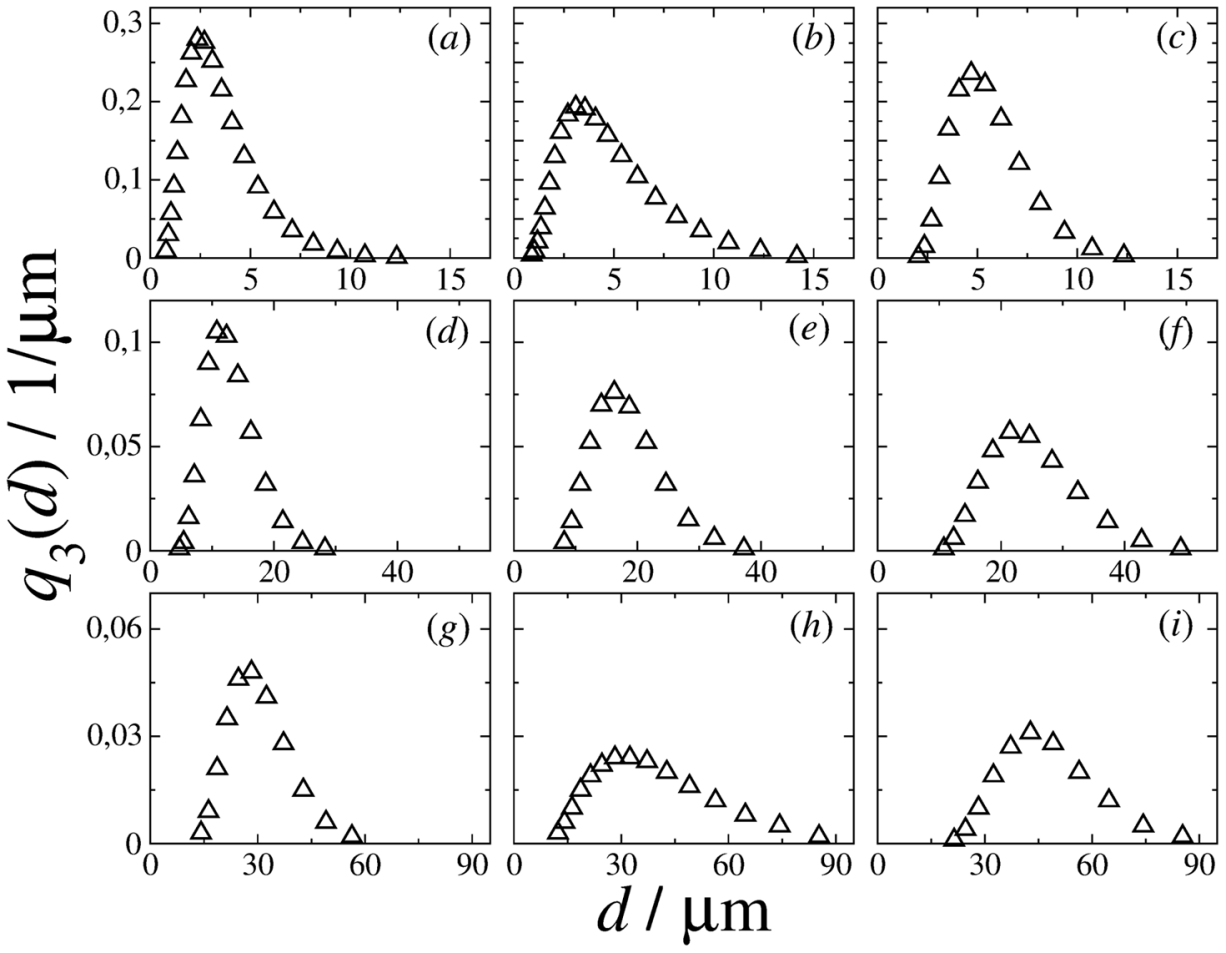
Experimental particle size distributions. The figure shows the volume density distributions *q*_3_ (see Eq. (1)) of the samples *a*–*i* used in the experiments. Each plot gives the volume density distribution *q*_3_(*d*) as a function of the particle diameter *d* in the sample.

**Figure 2:**
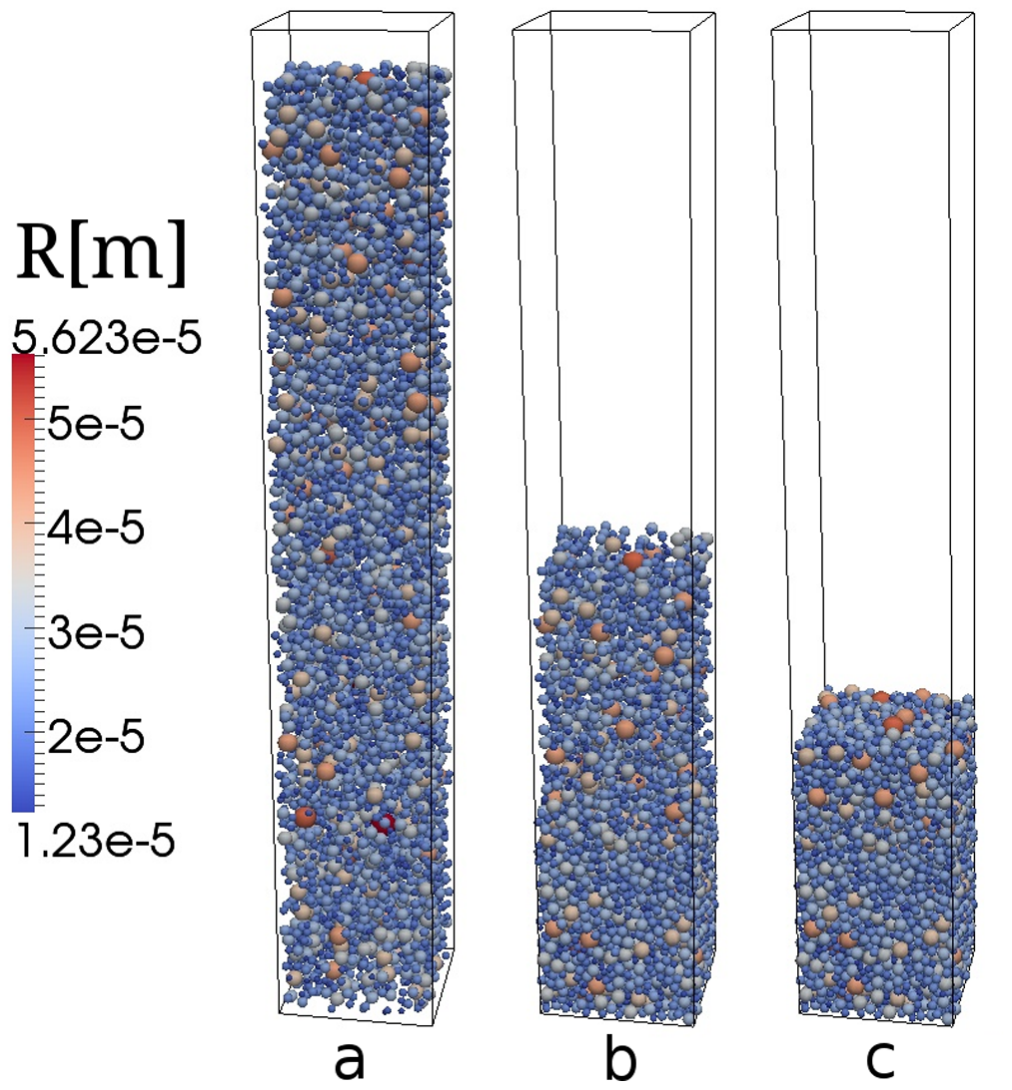
Numerical simulation of the powder packing. The figure displays a packing of 6172 cohesionless spherical particles of size distribution shown in Fig. 1i. The box size is *L_x_* = *L_y_* = 0.3351 mm (periodic boundary conditions) and *L_z_* = 4.2 mm. Figures a–c show snapshots at time (in milliseconds) 0, 20 and 140.

For the simulation we adopt the mass fractions of each particle size in the sample as obtained from the corresponding volume density distribution used in the experiment, Fig. 1. Silica glass particles are deposited in a rectangular box of lateral dimensions *L_x_* × *L_y_*, where *L_x_* = *L_y_* = 12 〈*d*〉, with 〈*d*〉 standing for the mean particle size, specific for each sample. We apply periodic boundary conditions in the directions *x* and *y* (Fig. 2). A frictional wall is placed at the floor, *z* = 0, while the height of the box (*L_z_*) is set large enough such as to produce packings with depth larger than 30 〈*d*〉. The equations used for computing the forces between particles and the frictional wall at the bottom are the same used for modeling particle-particle collisions where one of the contact partners is of infinite mass and radius. For particle-wall contacts we neglect attractive forces.

**Figure 3:**
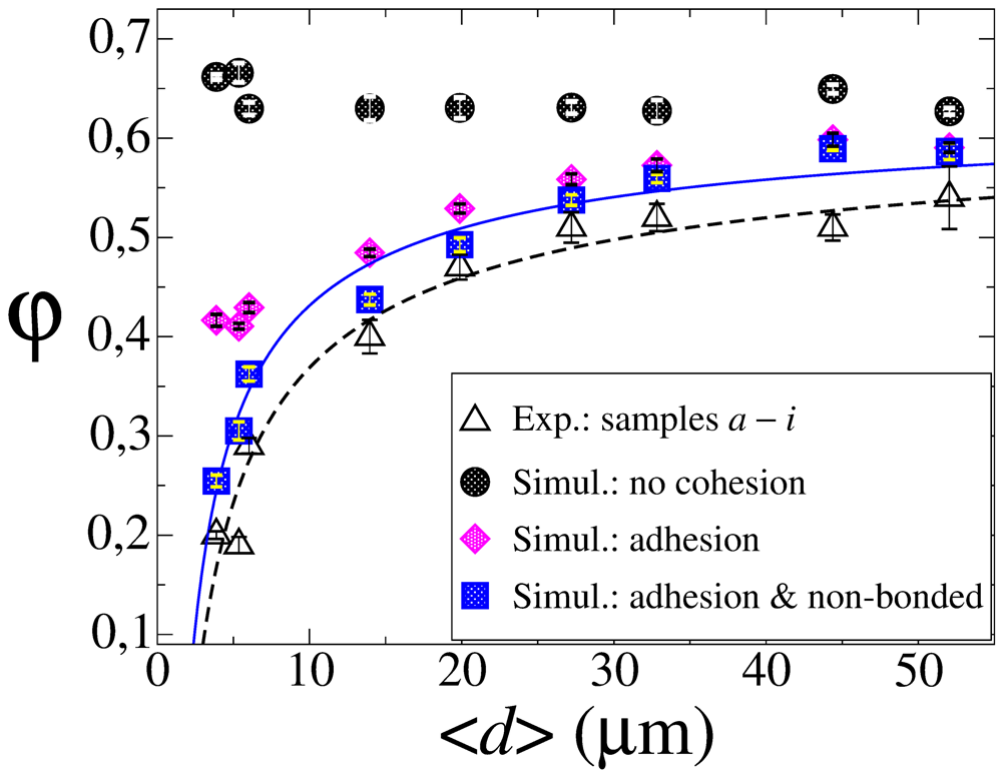
Packing fraction as a function of the average particle size. Empty symbols show experimental results for samples *a*–*i*, each corresponding to a different particle size distribution, specified by Fig. 1 and Tab. I. Results of the simulation are shown by filled symbols: Circles: no attractive forces; diamonds: with adhesion (JKR model); squares: with both adhesion and non-bonded van der Waals interactions. The lines show the best fit to the data using Eq. (12).

For initial conditions, we place the particles at random positions (initial space filling *φ*(0) ≈ 0.2) such that the particles do not touch one another (Fig. 2a). At time *t* = 0 the particles are released from rest and are deposited at the bottom due to gravity. The density of the sediment, *φ*, was computed after full relaxation, indicated by vanishing kinetic energy of the particles, via 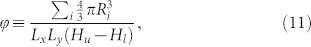
where the sum runs over all particles whose position is within the range (*H_l_*, *H_u_*) = (0.3, 0.7)*z*_max_ with *z*_max_ being the vertical position of the highest particle in the packing. We found that the value of *φ* obtained for a specific particle size distribution and inter-particle force model varies by a negligible amount over different realizations. Therefore, the values of *φ* presented in the following were obtained by dividing both lateral dimensions of the box in two equal parts and averaging over the 4 resulting boxes, whereas the corresponding standard deviation associated with the different simulation data is indicated by the error bars in Fig. 3.

Figure 3 shows the packing fraction for the samples *a*–*i* as obtained in the experiment and the simulations. In the experiment, we find nearly constant packing fraction, *φ*, for samples *f*–*i* where 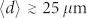 (see Figs. 1f–i) while for smaller particles *φ* decreases rapidly. Such a behavior of increasing porosity with decreasing particle size was also noted in previous experiments, and has been attributed to the tendency of fine particles to form tree- or chain-like packing structures as well as large agglomerates^1^.

In order to understand the effect of inter-particle forces on the packing properties and, thus, the dependence of the packing fraction on the average particle size, we performed three different groups of simulations, which are described below.

### Pure viscoelastic interaction

In the first group of simulations, the packings are produced by neglecting attractive interaction forces, resulting in almost constant packing fraction (filled circles in Fig. 3) in disagreement with the experimental data. In fact we observe even a small (statistically not significant) increase of the packing fraction for small average particle size. This effect may be understood since the samples of smallest average size reveal the largest width of the distribution (Figs. 1a,b and Tab. I). Therefore, the small particles can efficiently fill the pores between the large ones, thus leading to lower porosity values. The effect of pore filling in polydisperse packings has been extensively studied in the past and provides the physical mechanism exploited in the search for the optimal packing of non-cohesive granular materials^15^,^16^,^17^,^18^,^19^,^20^.

### Adhesive viscoelastic interaction

In the second group of simulations, the adhesive force model (Eq. (6)) is incorporated leading to a decrease of the packing fraction for all samples and in particular for small particles (filled diamonds in Fig. 3). Obviously, for small particles, adhesion becomes dominant over gravity and, thus, particles tend to stick to one another leading to higher porosity, resulting in a decay of the packing fraction with decreasing particle size. While the decay follows the experimental data for 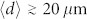, in agreement with earlier findings obtained for monosized particles^13^, for smaller particles the data disagree due to increasing importance of van der Waals forces neglected here. Moreover, for all particle sizes, there is a significant offset between the experimental and numerical data.

### Full model, including viscoelastic, adhesive, and van der Waals interaction

For the third group of simulations we applied the full model of particle interaction, that is Eqs. (6) and (9) (filled squares in Fig. 3). The resulting packing fraction agrees rather well with the experiment, as compared with the previous simulations where parts of the interaction force were neglected. In particular, for small particles, 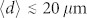, the packing fraction decreases rapidly, in agreement with the experiment. For larger 〈*d*〉, we obtain essentially the same packing density as found in simulations including only adhesive forces, since the ratio of van der Waals forces (which scale with *R*; cf. Eq. (9)) and particle weight (which scales with *R*^3^) decreases as 1/*R*^2^. Therefore, the contribution of van der Waals forces renders negligible as the particle size increases. The full line in Fig. 3 shows the best fit of the function 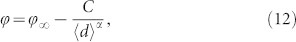
to the numerical data, where *φ*_∞_ ≈ 0.64 is the packing density in the limit of large particles, where attractive forces are negligible. In this limit the packing fraction approaches the value found for cohesionless particles (filled circles in Fig. 3). Table II shows the fit parameters, *C* and *α*, together with the corresponding values for the experimental data (dashed line in Fig. 3), being in good agreement.

Obviously, the function Eq. (12) cannot be universal as lim_〈*d*〉→0_ *φ* → −∞, however, for particle sizes in a certain range, 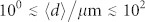 it describes the packing fraction rather well. Indeed, we have showed, for the first time, that both experimental and numerical data of the packing fraction of fine powders as a function of the average particle size can be fitted by a surprisingly simple expression (Eq. (12)), which contains only 2 fit parameters. However, certainly Eq. (12) is not unique since the values of the fit parameters depend on material properties, in particular on the cohesion energy density, *γ* which was not investigated here.

## Discussion

We studied the packing fraction, *φ*, of fine glass powders of given particle size distribution by means of DEM simulations in comparison with experimental results. Three sets of simulation were performed, assuming different particle interaction forces.

For pure viscoelastic interaction (set I) we obtain packing fraction almost independently of the mean particle size, 〈*d*〉, with a slight tendency of increasing *φ* for small particles which may be explained by geometric effects (pore filling). This behavior, *φ* ≈ const., agrees with the experiment for large particle size, 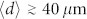 but disagrees for smaller particle size.

Simulations incorporating JKR-type adhesive forces (set II) reveal a clear decay of *φ* with decaying 〈*d*〉 and we obtain a good agreement with the experiment for 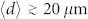 while the data diverge for smaller particles.

The third set of simulation assumed inter-particle forces due to viscoelastic, JKR-adhesive and non-bonded van der Waals interaction (set III) which finally allowed to reproduce the experimentally found packing fraction for the full interval 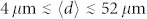.

Therefore, we conclude that for a predictive simulation of fine powder behavior, both adhesive and van der Waals forces are essential and should, thus, be considered in DEM simulations. Neglecting any of these contributions in simulation of fine powders may lead to unreliable results.

We believe that this result, obtained for the packing of fine glass powders will be of relevance also for other related systems^40^, in particular, when the system contains a significant fraction of small particles or a wide distribution of particle sizes. The present study should be now continued by performing a detailed investigation of the different packing structures obtained in experiments and simulations using different material properties and particle size distributions. Such an investigation could be conducted by using e.g. scattering or tomography to directly compare spatial correlations between experimental and modelled particles and to shed further light on the role of polydispersity on the packing behavior of the powder system.

We note that other factors may further influence the exact value of the packing fraction. In particular, experimental spherical particles generally display small variations from the perfect spherical shape, which can affect local properties of the packing. In order to account for rolling resistance of slightly non-spherical particles, the DEM simulations of Ref. [41], which used a linearized version of Eq. (9), included an empiric model for rolling friction. However, Ref. [41] did not consider the contribution of adhesive forces predicted by the JKR theory (see Eq. (6)). Indeed, recent DEM simulations of packings of monosized rods with adhesion forces modeled with the JKR theory^13^ suggested that a slight degree of non-sphericity should cause negligible change in the solid fraction of the bulk. For spherical particles, rolling friction plays a minor role for particle motion compared to sliding friction^42^. Further, the effect of adhesion on the bulk solid fraction is predicted to be greater the larger the particles' aspect ratios^13^. To simulate powder particles of irregular shapes, our model should be thus extended in order to incorporate the multisphere method (see e.g. Refs. [10, 13, 43] and references therein), while accounting for both adhesion and non-bonded van der Waals forces between the particles as described in the present work.

Moreover, attractive forces due to the formation of liquid bridges between the particles^39^ that have been neglected in the present simulations will also play an important role in the experiments since these were performed under ambient conditions in the laboratory at a relative humidity of around 40%. Indeed, the values of surface energy density and Hamaker constant used in the simulations are consistent with those applied in previous experiments under comparable experimental conditions^3^. However, while we have shown that a remarkable improvement in the quantitative agreement between numerical and experimental measurements of the solid fraction of fine polydisperse powders can be obtained by including adhesive and non-bonded van der Waals forces in DEM simulations, our study must be further improved in the future by inclusion of liquid bridges in the DEM simulation. This further extension of the DEM model certainly should further improve the quality of the agreement between numerical predictions and experimental results.

Experimental particles of different sizes may also have different friction coefficients, which may further affect the value of bulk packing fraction *φ*. However, the effect of *μ* on *φ* was shown in many previous DEM simulations to be negligibly small (less than 10%; see e.g. Refs. [44, 45]) compared to the strong dependence of *φ* on 〈*d*〉 found in our experiments (Fig. 3). Moreover, fluid friction on air is known to affect the deposition dynamics of fine particles^46^,^47^, however, clearly the cohesive properties of such particles dominate the static behavior of the bulk. Furthermore, it is well known that the solid fraction of a granular system may be influenced by the assembly procedure^10^,^48^. Therefore, our expression for predicting the packing fraction of powders as a function of the average particle size, Eq. (12), should be tested not only for different size distributions and material properties but also for different assembly procedures. Indeed, the bulk solid fraction of granular systems made of non-cohesional spherical particles is close to 0.64 (the random close packing^49^, consistent with our simulation results denoted by the black circles in Fig. 3), and is well reproducible by many different assembly procedures^50^,^51^. In contrast, numerical simulations^41^,^52^ show that the packing behavior of cohesive powders under compression may strongly depend on the preparation method. However, the aim of our work is to reproduce the specific conditions of the experiments, in which the powder is deposited into a recipient and is not subject to compression (see for instance previous DEM simulations of Refs. [7, 11, 13, 16, 44, 45]). Therefore, on the basis of the exposed above, we conclude that attractive particle interaction forces play a major role for the bulk solid fraction of fine polydisperse powders made of spherical particles, while additionally including the aforementioned factors should further improve the quantitative assessment of the packing behavior of cohesive granular systems.

## Methods

### Experimental details

Commercially available, spherically-shaped glass particles SiLibeads Type S (0–20 *μ*m, 0–50 *μ*m, 40–70 *μ*m, Sigmund Lindner GmbH) have been classified using a 50 ATP Turboplex air classifier (Hosokawa Alpine AG) to obtain glass powders of different particle size used in the experiment.

The particle size distributions of the samples *a*–*i* of glass powders have been obtained by laser diffraction particle sizing using a Mastersizer 2000/Hydro 2000S (Malvern). An aqueous suspension of the glass particles has been diluted as appropriate prior to measurement with deionized water. In each measurement, the respective scattering intensity raw data of the dispersed particles are collected by a detector array in dependency on the scattering angle and converted to particle size distributions by a Mie theory algorithm that is implemented in the Mastersizer 2000 software. As Mie scattering describes the scattering of electromagnetic radiation by a homogeneous sphere, reliable size distributions are obtained in the case of the almost transparent and spherical glass beads considered in this study. For evaluation a refractive index of 1.52 for the glass beads and 1.333 for water was used, respectively.

In order to determine the bulk density of packings we poured approx. 80 mL of the respective glass powder into a graduated cylinder using a funnel of a total nominal volume of 100 mL (resolution of 0.5 mL) and obtained the occupied volume from the filling height. The funnel has top and bottom diameters of 10 cm and 1.5 cm, respectively, and an angle of 60°, while the diameter of the cylinder is 3.5 cm. The experiments were performed with the funnel outlet at a constant height of about 12 cm from the recipient's bottom. For all samples, after the powder was poured from the funnel into the recipient, the measurements of the packing fraction were performed without applying any tapping or compression to the granular material. The bulk density was obtained by dividing the mass of the material as obtained using a lab balance (resolution 1 mg) by the occupied volume. The packing fraction, *φ*, is obtained by dividing the bulk density by the density of the solid. The results obtained for the packing fraction were found to vary only marginally over the 5 different experimental realizations (see standard deviation indicated by the error bars in Fig. 3).

### DEM

The integration was performed using LIGGGHTS^26^ which was extended to account for the attractive particle interaction forces (Eqs. (6) and (9)). The Young modulus and particle mass density are taken from the data sheet of the material used in the experiments. For the surface energy density of the beads we use a value which is typical for SiO_2_ and calculate the Hamaker constant using Eq. (10). Moreover, the Poisson's ratio of silica glass is used in the simulations (see e.g.^53^). For the friction coefficient *μ* we take a value that is typically used in simulations of granular materials (see e.g.^10^). The numerical values are given in Tab. III.

The integration time step Δ*t* must be small enough to accurately solve Newton's equations for the particle interaction. For undamped, non-adhesive collisions (*A*_n_ = *γ* = 0), the duration *T*_col_ of the collision can be estimated using the equation^22^, 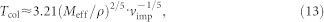
where *v*_imp_ is the impact velocity. Typically a timestep smaller than about *T*_col_/50 is recommended^54^. Thus, in order to determine the timestep, first we estimate, using Eq. (13), the collision time of the smallest particles in the particle size distribution using a reference impact velocity *v*_imp_ = 1.0 m/s (which is in fact an upper bound for the particle velocities observed in the simulations). Thereafter, we take Δ*t* about 1/50 of *T*_col_. For example, for the size distribution in Fig. 1i, the smallest value of particle diameter is *d* ≈ 20 *μ*m, for which we obtain *T*_col_ ≈ 62 ns and thus we choose Δ*t* ≈ 1.2 × 10^−9^ s.

### Solution of Eq. (8)

In this Section, we present the expression which we use to obtain the contact radius *a*(*ξ*). This contact radius is obtained by solving Eq. (8), which is a quartic equation in *a* of the form, 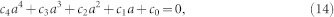
with 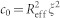, 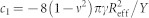, *c*_2_ = −2*R*_eff_*ξ*, *c*_3_ = 0 and *c*_4_ = 1. The discriminant of Eq. (14) reads, 
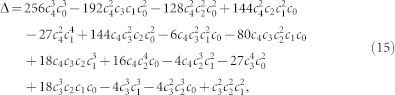
which becomes, by using the values of *c*_0_, …, *c*_4_ mentioned above, 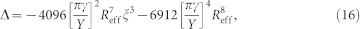
and is, thus, always negative. Therefore, Eq. (14) has 2 real roots and 2 imaginary roots. The solution *a*(*ξ*) which corresponds to the contact radius of the JKR model is the one that is larger than 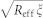 (the contact radius associated with the non-adhesive viscoelastic contact). Let us define the following quantities^55^, 
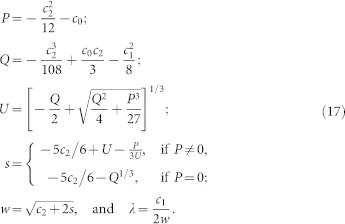
The real roots of Eq. (14) are 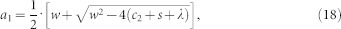

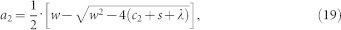
whereas the root that is larger than 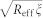 is the root *a*_1_. Therefore, the solution for the contact radius from Eq. (8) is *a*(*ξ*) = *a*_1_, Eq. (18).

## Author Contributions

E.J.R.P. performed the numerical simulations and wrote the paper with T.P. J.S. and C.B. performed the experiments. J.S., K.W. and W.P. discussed the results with E.J.R.P. and T.P. and added content to the manuscript.

## Figures and Tables

**Table 1:** Summary of the powder characteristics of samples *a*–*i*: Quantities *d*_1,3_, *d*_50,3_ and *d*_99,3_; mean particle size 〈*d*〉, and obtained packing fraction *φ*. Samples *a*–*i* correspond to the subplots of Fig. 1

sample	*d*_1,3_/*μ*m	*d*_50,3_/*μ*m	*d*_99,3_/*μ*m	〈*d*〉/*μ*m	*φ*
a	1.05	3.22	9.51	3.88	0.20
b	1.40	4.47	12.33	5.36	0.19
c	2.65	5.35	10.79	6.03	0.29
d	6.31	12.49	23.89	13.96	0.40
e	9.18	17.81	33.44	19.87	0.47
f	2.87	24.47	44.94	27.21	0.51
g	15.78	29.56	53.74	32.83	0.52
h	14.15	38.37	89.13	44.38	0.51
i	25.27	46.85	85.05	52.04	0.54

**Table 2:** Fit parameters of Eq. (12) for the experimental and numerical data and the corresponding correlation coefficient

	*C*	*α*	correlation coeff.
experiment	1.049	0.587	0.981
simulation	0.990	0.676	0.987

**Table 3:** Numerical values of the parameters used in the simulations

parameter	symbol	value
particle material density		2500 kg/m^3^
Young's modulus	*Y*	63 GPa
Poisson's ratio	*ν*	0.24
Coulomb's friction coefficient	*μ*	0.50
surface energy density	*γ*	0.05 J/m^2^
Hamaker constant	*A_H_*	10^−19^ J
